# MicroRNA-31 Regulates Expression of *Wntless* in Both *Drosophila melanogaster* and Human Oral Cancer Cells

**DOI:** 10.3390/ijms21197232

**Published:** 2020-09-30

**Authors:** Ji Eun Jung, Joo Young Lee, In Ryoung Kim, Sang Mee Park, Ji Wan Kang, Yun Hak Kim, Hae Ryoun Park, Ji Hye Lee

**Affiliations:** 1 Department of Life Science in Dentistry, School of Dentistry, Pusan National University, Yangsan 50612, Korea; totoro4640@hanmail.net (J.E.J.); parkhr@pusan.ac.kr (H.R.P.); 2 BK21Plus Project, School of Dentistry, Pusan National University, Yangsan 50612, Korea; 3 Dental and Life Science Institute, Pusan National University, Yangsan 50612, Korea; prdm16@gmail.com; 4 Department of Anatomy, School of Dentistry, Pusan National University, Yangsan 50612, Korea; biowool@pusan.ac.kr; 5 Department of Oral Pathology, School of Dentistry, Pusan National University, Yangsan 50612, Korea; psm0322@naver.com; 6 Interdisciplinary Program of Genomic Science, Pusan National University, Yangsan 50612, Korea; jwkang3929@naver.com; 7 Department of Anatomy, Department of Biomedical Informatics, School of Medicine, Pusan National University, Yangsan 50612, Korea; yunhak10510@pusan.ac.kr

**Keywords:** microRNA-31, *Drosophila melanogaster*, oral cancer, *Wntless*, Wnt pathway, mammal-to-*Drosophila*-to-mammal approach

## Abstract

Recent comparative studies have indicated distinct expression profiles of short, non-coding microRNAs (miRNAs) in various types of cancer, including oral squamous cell carcinoma (OSCC). In this study, we employed a hybrid approach using *Drosophila melanogaster* as well as OSCC cell lines to validate putative targets of oral cancer-related miRNAs both in vivo and in vitro. Following overexpression of *Drosophila* miR-31, we found a significant decrease in the size of the imaginal wing discs and downregulation of a subset of putative targets, including *wntless* (*wls*), an important regulator of the Wnt signaling pathway. Parallel experiments performed in OSCC cells have also confirmed a similar miR-31-dependent regulation of human *WLS* that was not initially predicted as targets of human miR-31. Furthermore, we found subsequent downregulation of *cyclin D1* and *c-MYC*, two of the main transcriptional targets of Wnt signaling, suggesting a potential role of miR-31 in regulating the cell cycle and proliferation of OSCC cells. Taken together, our *Drosophila*-based in vivo system in conjunction with the human in vitro platform will thus provide a novel insight into a mammal-to-*Drosophila*-to-mammal approach to validate putative targets of human miRNA and to better understand the miRNA-target relationships that play an important role in the pathophysiology of oral cancer.

## 1. Introduction

A perturbation of Wnt signaling and its role in segment polarity was first demonstrated in *Drosophila* mutants of a *wingless* allele (*wg^1^*) [1], and subsequent gene cloning of its sequence [2] revealed a significant degree of homology to human and murine *INT-1* [3,4]. Wnt proteins play an important role in various aspects of development, including cellular differentiation, proliferation, apoptosis and migration [5]. Following their release to the extracellular environment, Wnt proteins trigger receptor-mediated intracellular signaling in the neighboring cells via two distinct modes: (1) the “canonical signaling pathway” through which intracellular Wnt can modulate the activity of β-catenin and subsequently regulate intranuclear transcription of multiple targets in combination with T-cell factor (TCF) and lymphoid enhancer-binding factor (LEF); or (2) the “non-canonical signaling pathway” that can be subdivided into two distinct pathways, the Wnt-dependent planar cell polarity and the Wnt/Ca^2+^ pathway, both of which can modulate transcription of target genes via protein kinase C or c-Jun N-terminal kinase signaling [6].

In addition to its endogenous activity during development, abnormal regulation of Wnt signaling has been implicated in a number of human diseases, including cancer. For instance, enhanced signaling of the Wnt/β-catenin pathway is one of the most common cellular hallmarks detected in the majority of cancers [7,8]. Initiation of canonical Wnt signaling triggers transcription of several effector genes important for tumorigenesis and cancer progression, including c-MYC and cyclin D1 [9]. The activity of the canonical Wnt signaling pathway has also been linked to the development of chemoresistance in various types of cancer, including breast, lung and pancreatic cancer [10]. In addition, dysregulation of non-canonical Wnt signaling may also contribute to tumorigenesis and cancer progression via metabolic and inflammatory reprogramming in a subset of cancer [11,12]. Recent reports suggest that malignant tumors occurring in the oral cavity may also be influenced by these Wnt signaling pathways [13], as demonstrated by the dysregulation of the soluble frizzled receptor protein family, a representative Wnt receptor, in oral squamous cell carcinoma (OSCC) cells [14]. With these results together, targeting of Wnt signaling may thus be an effective alternative to the current remedies to tackle oral cancer [15].

Recent reports have also suggested microRNA (miRNA)-dependent regulation of putative oncogenes and tumor suppressors, including Wnt signaling molecules, as a potential mechanism underlying tumorigenesis and progression of oral cancer [16]. miRNAs represent a class of short, non-coding RNAs that regulate translation and degradation of target mRNAs [17]. The majority of recent studies have aimed to display different expression profiles of individual miRNAs between normal and oral cancer tissues as well as OSCC cell lines [18,19]. These attempts have yielded a list of candidate miRNAs potentially linked to oral cancer, including miR-21, miR-34, miR-133 and miR-203 [20]. However, with miRNAs of the same family functioning as either oncogenic or tumor-suppressive, depending upon the nature of each malignancy, inconsistent recent findings made it difficult to draw a solid conclusion about the role of a specific miRNA in tumorigenesis and progression of oral cancer. Furthermore, the current approach of miRNA profiling has failed to provide further insights into a causal relationship between dysregulation of specific miRNAs and the pathophysiology of oral cancer. It should also be noted that each miRNA in general may potentially regulate a few hundred target mRNAs relevant to oral cancer. Therefore, experimental validation of putative targets of each miRNA is crucial to dissect the contribution of the specific targeting of proto-oncogenes or tumor suppressors by individual miRNA to tumorigenesis and cancer progression.

The putative targets of OSCC-related miRNAs deduced from recent in vitro studies encompass components of multiple signaling pathways, including Wnt described above. For instance, miR-21 and miR-148a were reported to target *Dickkopf-2* and *Wnt10b* in vitro, respectively, thus allowing the canonical Wnt signaling pathway to be further activated to promote invasiveness of OSCC cells and oral cancer-associated fibroblasts [21,22]. In addition, miR-218-dependent regulation of the Wnt signaling pathway has also been implicated in the development of chemoresistance in oral cancer cells [23]. Despite the limited availability, these studies strongly suggest miRNA-dependent regulation of the Wnt pathway as an important mechanism to control the formation and progression of oral cancer in vitro. However, whether similar functional networks between the miRNAs and Wnt pathway operate in vivo remains poorly understood.

In this study, we adopted an experimental animal model, *Drosophila melanogaster*, in order to investigate the functional relationship between individual miRNAs and their putative targets in vivo. While experimental paradigms for target validation in vivo have mostly utilized mouse models, transgenic approaches in such models often require extended periods of experimental confirmation, hindering fast and efficient validation of putative targets. In this regard, *Drosophila* may pose itself as an attractive alternative model with high homology for human disease-related genes, but with relevant signaling pathways of less redundancy [24]. In addition, extremely sophisticated genetic tools employed in the field of *Drosophila* allows manipulation of each gene from the level of single cells to the entire organism. Taking advantages of this model organism, we investigated the relationship between individual miRNAs and their putative targets in vivo, by manipulating the expression of each miRNA in a specific *Drosophila* tissue, followed by monitoring the transcript level of the targets. Among the miRNAs screened for their tumorigenic potential, overexpression of *Drosophila* miR-31 (dme-miR-31) induced a significant reduction in tissue growth, suggesting its function as a tumor suppressor. Importantly, the level of *wntless* (*wls*) mRNA, one of its putative targets and an important regulator of Wnt signaling pathway, was significantly downregulated in *Drosophila* following overexpression of miR-31. Despite the remotely predicted targeting of *WLS* suggested by the human database, our observation in human oral cancer cells following transfection of miR-31 indicated an apparent reduction in *WLS* mRNA and its protein product as well as a subset of downstream effectors of Wnt signaling. These results thus strongly support the idea of conserved targeting of *WLS* by miR-31 both in vivo and in vitro.

Taken together, our study demonstrates an adoption of *Drosophila* as a valuable in vivo platform for aiding functional validation of human miRNA-dependent regulation of its putative targets projected by computational analyses. Furthermore, a combinatorial approach using both *Drosophila* and human OSCC cells in our study demonstrates a successful example of the mammal-to-*Drosophila*-to-mammal paradigm to study the pathophysiology of oral cancer.

## 2. Result

### 2.1. miR-31 is Differentially Regulated in Various Types of Malignant Tumors, Including Oral Cancer

The significance of microRNAs (miRNAs) in development and progression of oral cancer has been a focus of recent efforts to better understand its pathophysiology. A number of comparative studies revealed differential expression profiles of multiple miRNAs when examined in either cultured oral squamous cell carcinoma (OSCC) cell lines or tumor tissues obtained from patients (Table 1). In line with these results, an approach to dissect the contributions of individual miRNAs to tumorigenesis and disease progression can be beneficial for development of targeted diagnostic and treatment options to cure oral cancer. Among these microRNAs, we were specifically interested in miR-31, of which function and contribution to the development of malignant tumors, including OSCC, remains relatively controversial and obscure.

**Figure 1:**
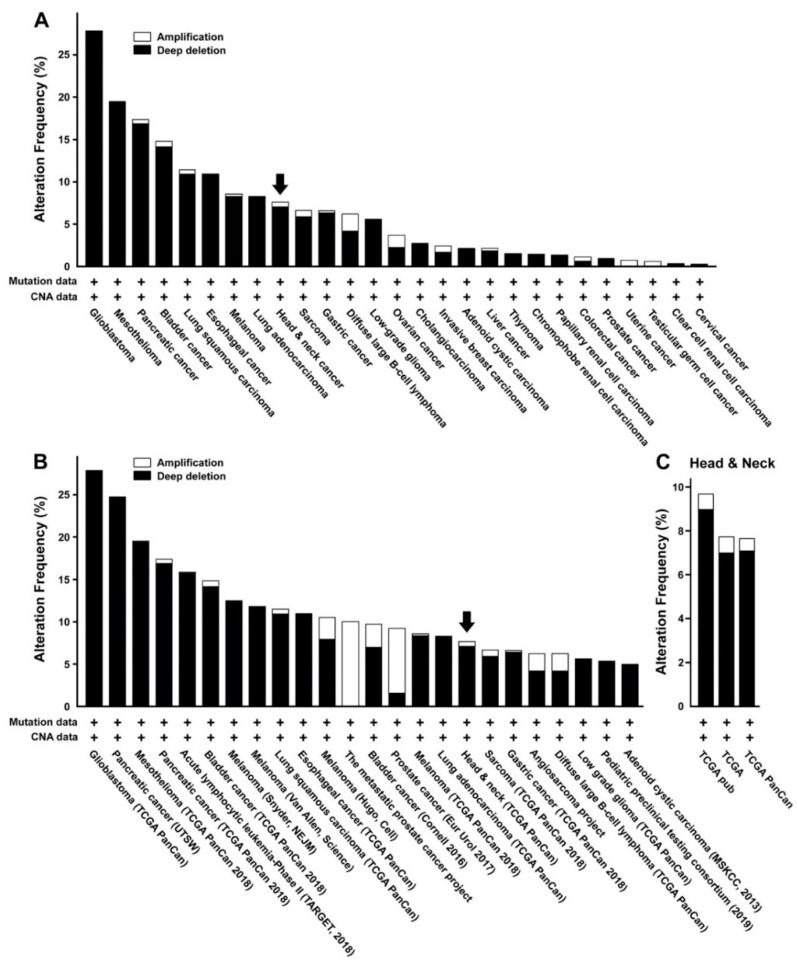
Expression of miR-31 is altered in head and neck cancer, including oral cancer, as well as in other malignant tumors. (**A**,**B**) The pooled TCGA PanCancer Atlas ((**A**), 10,967 samples from 10,953 patients) and curated non-redundant data (**B**), 47,005 samples from 44,597 patients) available from the cBio portal for cancer genomics [84,85] are shown for a variety of malignant tumors. The fraction of head and neck cancer samples is indicated as arrows in each plot. (**C**) All available data of head and neck cancer from the cBio portal are pooled together, with 1934 samples from 1932 patients [84,85].

When probed for its alteration in various types of tumor cells or tissues from previous reports and public genomic datasets, we were able to retrieve highly variable expression profiles of miR-31, depending upon the identity of the malignant tumors (Figure 1A,B, Table 2). A few studies on oral cancer indicated presumed upregulation of miR-31 in cultured cell lines or tumor tissues (Table 1). However, a retrieval from a larger pooled genome database revealed mixed outcomes among oral cancer patients, with mostly downregulated than upregulated miR-31 in the majority of cases (Figure 1C). These conflicting results make it hard to draw a solid conclusion about miR-31 being either an oncomir or a tumor suppressor in case of oral cancer. In addition, the molecular target of miR-31 and its significance with respect to the pathophysiology of oral cancer in vivo remain poorly understood.

### 2.2. Overexpression of Drosophila miR-31a and miR-31b Induced Changes in the Size of Wing Discs and the Levels of Putative Target mRNAs

**Figure 2:**
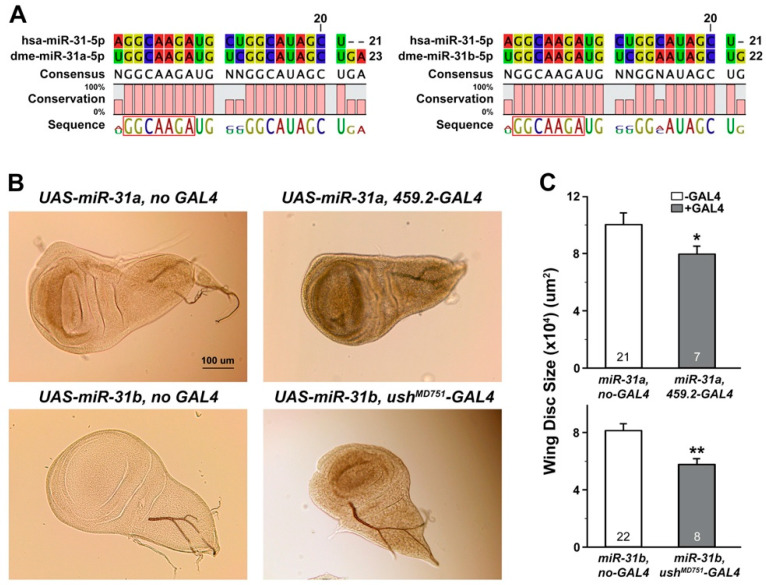
Overexpression of *Drosophila* miR-31a and miR-31b induced a significant reduction in the size of the imaginal wing discs. (**A**) A sequence comparison is shown among human and *Drosophila* miRNA-31s (hsa-miR-31-5p, dme-miR-31a-5p and dme-miR-31b-5p). The conserved seed sequence among them is indicated in boxes. (**B**) Representative images of the wing discs are shown for each genotype indicated, with control discs on the left panels and the ones with overexpression of miR-31 on the right. Scale bar, 100 μm. (**C**) The size of the wing discs is compared among the genotypes indicated. Note that the data obtained from two independent lines of miR-31a transgenes (#1 and #6) are pooled together due to their similarity. The number of discs examined is indicated in each column. Mean ± SEM indicated. * *p* < 0.05 and ** *p* < 0.01.

Previous screens on differential expression of miR-31 in oral cancer cells or tissues were often concluded with suggestive putative targets [19,86], most of which were retrieved based upon available prediction platforms, including TargetScan (http://www.targetscan.org/vert_72) and PicTar (https://pictar.mdc-berlin.de). However, whether these putative targets were indeed to be regulated by miR-31 remains poorly demonstrated in experimental settings. In this study, we employed a novel approach to accomplish both in vivo and in vitro validations of the miR-31-target relationships, using a well-established in vivo model system, *Drosophila melanogaster*, as well as widely used OSCC cell lines in culture. Versatility of *Drosophila* as a model to explore the functional interaction between miR-31 and its putative targets was considered sufficient, based upon the presence of a conserved seed sequence within mature *Drosophila* miR-31 (dme-miR-31a and dme-miR-31b) (Figure 2A) as well as a significant degree of homology shared between human and *Drosophila* proteins [24].

To screen for mRNAs regulated by miR-31 in *Drosophila*, we adopted an approach to overexpress miR-31 in the epithelial tissues that would emulate target mRNAs being regulated by human miR-31 in oral cancer of an epithelial origin. For this purpose, larval imaginal wing discs were chosen for the site of overexpression of miR-31, as these structure metamorphosing into adult wings in future are largely composed of epithelial cells and have been proven to be a useful platform to investigate the function of tumor suppressors and oncogenes [87,88]. As our initial attempt to monitor the level of overexpression with miR-31 sequence-specific primers failed to detect positive signals, we performed a RT-PCR analysis using a primer set against the luciferase sequence inserted within the UAS-miR-31 constructs (see Materials and Methods). Indeed, tissue-specific overexpression of miR-31 using the well-established GAL4-UAS system [89] was confirmed by the presence of amplified signals only in the presence of the GAL4 counterpart (Supplementary Materials Figure S1). Importantly, overexpression of two independent miR-31 transgenes (dme-miiR-31a and dme-miR-31b) in different parts of the larval wing discs resulted in a significant reduction in the size of the wing discs compared to its controls (Figure 2B,C), suggesting a potentially suppressive role of miR-31 in tissue growth. Such an activity of miR-31 we observed appears inconsistent with recent reports that demonstrated its upregulation in cultured oral cancer cells and tumor tissues [28,29,30,31], but in line with the pooled cancer genome analyses of head and neck cancers, including OSCC (Figure 1C). It should be noted that the previous studies have mostly focused on deciphering the expression profiles of miR-31, without further intervention involving experimental manipulation in vivo.

### 2.3. Overexpression of Drosophila miR-31a Reduced the Transcript Level of a Predicted Target, WLS

As stated above, our current understanding of how miR-31 could influence the formation of oral cancer and its progression has been restricted to a computational algorithm-based prediction of the molecular targets regulated by the activity of miR-31. To our best knowledge, the majority of previous studies on miR-31 often lack experimental evidence to delineate such presumptive relationships between miR-31 and its putative targets.

**Figure 3:**
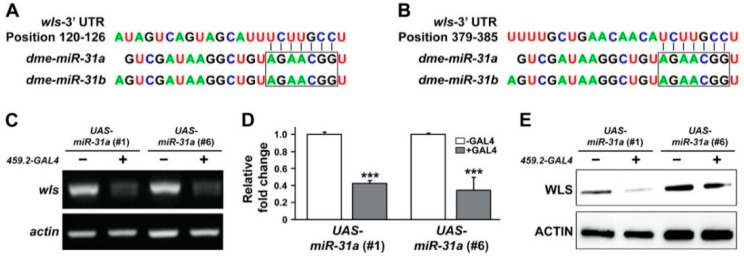
Overexpression of *Drosophila* miR-31a resulted in a significant reduction in the transcript level of a predicted target, *wntless (wls).* (**A**,**B**) The potential targeting sites within the 3′ UTR region of *wls* mRNA are identified with lines to the seed sequences of miR-31a and miR-31b (boxes). (**C**,**D**) The levels of *wls* mRNAs, a putative target of miR-31a, monitored from RT-PCR (**C**) and qRT-PCR analyses (**D**) are shown for each genotype indicated. Mean ± SEM indicated. *** *p* < 0.001. (**E**) The representative result of a Western blot analysis is shown to compare the levels of WLS protein extracted from the wing discs with and without overexpression of miR-31a. Note that the data obtained from two independent lines of miR-31a transgenes (#1 and #6) are shown separately to confirm the similarity of their phenotypes.

With the findings of miR-31-induced suppression of tissue growth (Figure 1), we aimed to validate the miR-31-dependent regulation of its putative targets in *Drosophila*. To accomplish this goal, putative targets were first retrieved using TargetScanFly (release 6.2 and 7.2, in 2012 and 2018, respectively; Figure S2). Among the suggested targets (Table S1), those with significant homology to human counterparts as well as relevant function to growth control were then selected and screened for changes in its transcript levels following overexpression of miR-31. Such candidates screened included *Drosophila wntless (wls)*, of which 3′-UTR was presumably targeted by both miR-31a and miR-31b (positions 120-126 and 379-385, as conserved and poorly conserved sites, respectively) (Figure 3A,B). Human ortholog of *Drosophila wls*, also known as *G-protein-bound receptors 177* (*GPR177*), encodes a transmembrane protein that controls trafficking of Wnt, thus playing an important role in Wnt-dependent regulation of tissue growth [90,91].

When an RT-PCR analysis was performed in larval wing discs overexpressing two independent miR-31s (miR-31a, #1 and #6), we found a significant reduction in the transcript level of *wls* (Figure 3C). A similar conclusion could also be drawn from the quantitative measurement of *wls* mRNA (Figure 3D). Downregulation of *wls* transcripts by overexpression of miR-31 was then translated into a lower level of protein products, as shown in a Western blot analysis (Figure 3E). Taken together, these results provide a solid in vivo evidence to experimentally validate the miR-31-dependent regulation of its putative target, *wls*. To our best knowledge, this is the first report that provides an experimental link between miR-31 and *wls* in vivo.

### 2.4. Overexpression of miR-31 in Human Oral Cancer Cells Induced Downregulation of Human WLS

Our data derived from *Drosophila* strongly suggest miR-31-dependent regulation of *wls* important for the Wnt signaling pathway. Based upon the conserved seed sequence between human and *Drosophila* miR-31s (Figure 2A), we screened for putative targets of human miR-31 (hsa-miR-31) using TargetScanHuman (release 6.2 and 7.2 in 2012 and 2018, respectively). Interestingly, *WLS* (or *GPR177*) was not listed as top candidates for miR-31-dependent regulation (Table S2) and listed as a low-ranked target only in a single prediction platform (DIANA tools, TarBase v.8). Such discrepancy prompted us to investigate whether the miR-31–*wls* relationship unraveled in *Drosophila* would also hold true in human oral cancer cells.

To address this issue, a potential targeting of *WLS* mRNA by human miR-31 was probed with a luciferase assay, by transient transfection of a target sequence within the *WLS* gene fused with the luciferase sequence as well as double-stranded miR-31 mimics containing the sequence of mature miR-31 into OSCC cells. A putative targeting site predicted by the aforementioned TarBase was found within the coding sequence of WLS far upstream of its 3′-UTR. This prompted us to search for other putative biding sites near or within 3′-UTR. It has been suggested that the efficacy of the miRNA-dependent targeting of its putative targets may vary depending upon the location of the target sites within 3′-UTR [92]. As a part of our effort to search the putative targeting sites, we first narrowed our search for the sites residing within 2000 nucleotides just following the last exon for two *WLS* variants. This attempt resulted in a list of candidates with a partial match to the seed sequence of miR-31. Despite the lack of complete seed match (Figure S3A), the luciferase activity of the OSCC cells transfected with a candidate construct was significantly reduced following transfection of miR-31 (Figure S3B). Such a trend was then reversed by co-transfection of a miR-31 inhibitor, an oligonucleotide carrying a complementary sequence against mature miR-31 (Figure S3B), raising the possibility of potential targeting of *WLS* by human miR-31.

**Figure 4:**
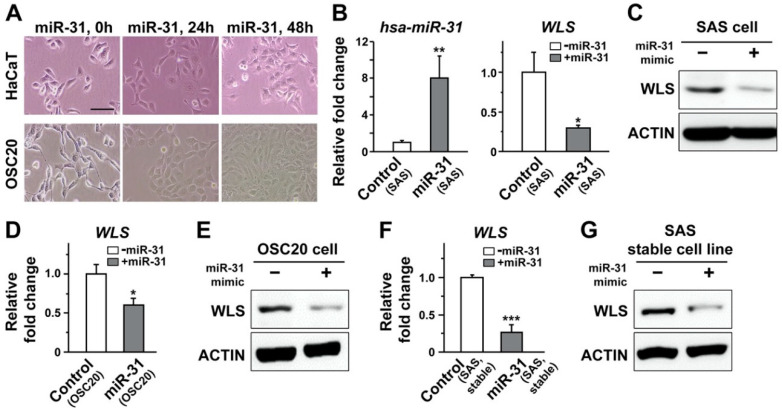
Overexpression of human miR-31 suppressed the transcript level of WLS, its putative target in oral cancer cells. (**A**) Representative images of HaCaT and OSC20 cells are shown following transient transfection of a miR-31 mimic, an oligonucleotide sequence resembling the mature miR-31, visualizing the effects of miR-31 on cell morphology. Scale bar, 50 µm. (**B**) The transcript levels of miR-31 (left) and its target, *WLS* (right), are shown before (control) and after transfection of miR-31 mimics (miR-31) in SAS cells. Mean ± SEM indicated. * *p* < 0.05 and ** *p* < 0.01. (**C**) The protein levels of WLS before and after transfection of the miR-31 mimics are compared in SAS cells. (**D**,**E**). The transcript and protein levels of *WLS* are indicated before and after transfection of miR-31 mimics in OSC20 cells. Mean ± SEM indicated. * *p* < 0.05. (**F**,**G**). As an alternative to transient transfection of the miR-31 mimics, the expression of WLS is quantified at the level of mRNA (**F**) and its protein product (**G**) in a SAS cell line stably expressing mature miR-31. Mean ± SEM indicated. *** *p* < 0.001.

We then quantified the level of *WLS* mRNA in representative OSCC cell lines, including SAS and OSC20, using a qRT-PCR analysis. The endogenous expression of miR-31 was estimated low to moderate at most, as presumed by relatively higher Ct value ranging from 33 to 36 cycles when compared to the values for other probes measured in the same experiments. Therefore, double-stranded RNA oligonucleotides resembling the sequence of mature miR-31 (miR-31 mimic) were transfected into these OSCC cells to improve the visibility of its potential regulation of WLS in subsequent analyses (Figure 4A). Importantly, a successful transfection of miR-31 mimic in SAS cells, confirmed by increased amount of hsa-miR-31 in lysates, led to a significant reduction in the level of *WLS* transcripts (Figure 4B). A similar trend of reduction was also evident when the level of WLS protein was assayed with a Western blot analysis (Figure 4C). In line with these results, an independent OSCC cell line, OSC20 cells, following a transfection of miR-31 mimic displayed a prominent reduction in both *WLS* transcripts and protein products (Figure 4D,E). As an alternative approach to enhance expression of miR-31, the OSCC cell lines with stable expression of miR-31 were established, with a varying range of overexpression around 6-fold or so. Consistent with those transfected with miR-31, the OSCC cell lines stably expressing miR-31 also exhibited significant downregulation of the *WLS* transcripts and protein products (Figure 4F,G; Figure S4), further confirming the miR-31-dependent regulation of human *WLS* that had not been preferentially projected by the majority of prediction platforms.

### 2.5. Overexpression of miR-31 in Human Oral Cancer Cells Induced Downregulation of Cyclin D1 and c-MYC

As WLS controls Wnt trafficking to induce activation of downstream effectors [90,91], we examined whether the expression of these effectors would also be altered in OSCC cells following transfection of miR-31 mimic. Our results indicated that the overall levels of the canonical Wnt signaling molecules, including Wnt3a and its downstream effectors such as GSK3β and β-catenin, were not significantly altered following enhanced expression of miR-31, with varying expression profiles in multiple cultures, thus making it hard for us to draw a solid conclusion (Figure S5A). Consistently, there was only a minimal change in the levels of these downstream effectors in OSCC cells stably expressing miR-31 (Figure S5B). In our *Drosophila* model, overexpression of miR-31 induced significant suppression of tissue growth (Figure 2), a phenomenon that could still reflect modifications in the activity of the Wnt signaling pathway, even without significantly altered expression of individual pathway components. As a part of our effort to unravel functional consequences of altered Wnt signaling relevant to miR-31-dependent regulation of WLS, the levels of Wnt signaling targets downstream of GSK3β and β-catenin were further monitored in OSCC cells.

**Figure 5:**
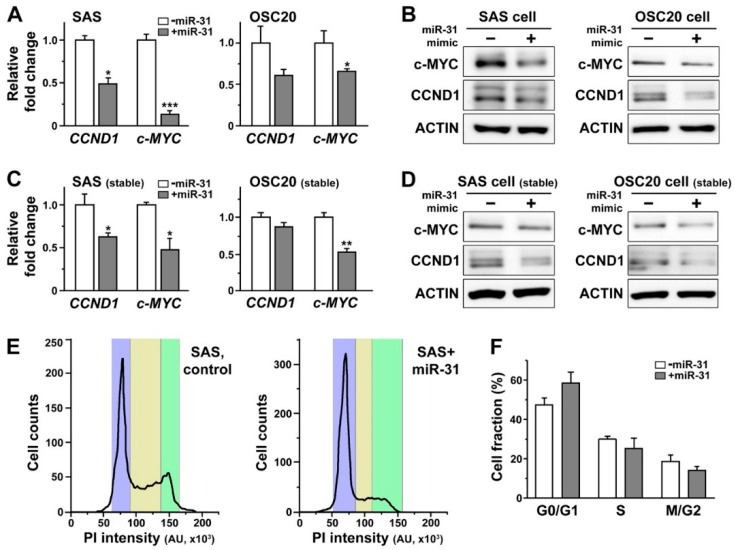
Overexpression of miR-31 in OSCC cells resulted in a significant down regulation of cyclin D1 (CCND1) and c-MYC and a shift in cell cycle from M-G2 to G0 phase. (**A**) The levels of *CCND1* and c-*MYC* mRNAs are monitored in SAS and OSC20 cells with and without transfection of miR-31 mimics. Mean ± SEM indicated. * *p* < 0.05 and *** *p* < 0.001. (**B**) The protein products of *CCND1* and *c-MYC* in SAS and OSC20 cells are visualized by a Western blot analysis with and without transfection of the miR-31 mimics. (**C**) The mRNA levels of *CCND1* and c-*MYC* are shown for SAS and OSC20 cells stably expressing mature miR-31. Mean ± SEM indicated. * *p* < 0.05 and ** *p* < 0.01. (**D**) The protein products of *CCND1* and *c-MYC* are visualized by a Western blot analysis in SAS and OSC20 cells stably expressing mature miR-31. (**E**) The profiles of propidium iodide staining of SAS cells with and without stable expression of miR-31 are shown to visualize the fraction of cell populations at different phases of the cell cycle. The blue, yellow and green sections each correspond to the G0/G1, S and M/G2 phase, respectively. (**F**) The cell fractions at different cell cycle phases are quantified in SAS cells with and without stable expression of miR-31.

When the miR-31 mimic was transiently expressed in SAS and OSC20 cells, we found a noticeable decrease in the levels of *cyclin D1* (*CCND1*) and c-*MYC* mRNAs (Figure 5A), two of the most common targets of the canonical Wnt signaling pathway that have been implicated in oral carcinogenesis [93]. Such transcriptional regulation of *CCND1* and *c-MYC* by miR-31 was further translated into a reduced amount of their protein products, as shown in the Western blot analysis (Figure 5B). In line with these results, stable expression of miR-31 in OSCC cells led to consistent decreases in both *CCND1* and *c-MYC* transcripts as well as their protein products (Figure 5C,D). It is important to note that *CCND1* and *c-MYC* are the classic proto-oncogenes involved in regulation of cell cycle and proliferation, thus playing a critical role in development of various types of malignant tumors [94]. Considering the significant downregulation of these proto-oncogenes, it is plausible to predict corresponding miR-31-induced changes in cell cycles of OSCC cells. Indeed, we found a slight shift in the ratio of OSCC cells in the G0/G1 phase over those in the M/G2 phase following stable expression of miR-31 in a subset of SAS cells (Figure 5E), with a similar trend in the pooled data detected, albeit a lack of statistical difference (Figure 5F). These data together may thus suggest a potential cell cycle arrest, preventing these cells from proceeding to further proliferation.

## 3. Discussion

Despite recent advances in research and treatment remedies, the 5-year survival rate of patients suffering from oral cancer, constituting 3.8% of all cancer cases and 3.6% of cancer mortality in 2012 [95], remains relatively low around 50% compared to other malignancies [96]. Besides, the incidence rate of oral cancer in recent years has significantly risen among the population younger than 45 years worldwide [97], requiring further effort to better understand its pathophysiology and to provide more effective treatment remedies. Meanwhile, dysfunction of microRNAs (miRNAs), one of the most abundant short non-coding RNAs, has been considered critical in development of a number of pathologic conditions, including various malignancies [98,99]. Recent findings on oral cancer also address abnormal regulation of miRNAs as potential molecular mechanisms that can be reversely engineered to develop novel therapeutic options (Table 1). In the hope of achieving this goal, a majority of the initial studies have focused upon profiling of differential levels of miRNAs either between normal and disease states in oral cancer-related case-control studies or between normal and cancer cell lines in vitro. The outcomes of these studies have indeed yielded a list of presumably dysregulated miRNAs (Table 1) and their putative targets predicted by computational algorithms incorporated in the public database [19]. However, one should be aware of false positive targets predicted by computational methods [100]. The next crucial step would be then experimental validation of these targets, either at the cellular or tissue level, to fully understand the role of a specific miRNA in the pathophysiology of oral cancer. So far, this validation step has been mostly performed in vitro, including demonstration of miRNA binding to the 3′-UTR region of its target.

In this study, we propose a hybrid platform combining both in vitro and in vivo systems for evaluation of the miRNA-target relationship relevant to oral cancer. In addition to the human oral squamous cell carcinoma (OSCC) cell lines representing an in vitro platform, we brought in *Drosophila melanogaster*, a well-established in vivo model system with relatively high homology to human disease-related proteins [24]. It is also important to note that *Drosophila melanogaster* is estimated to have 258 precursors as well as 466 mature miRNAs in the genome (miRbase, Release 22.1), most of which share significant sequence homology to their human counterparts [101]. These features together could make *Drosophila melanogaster* an attractive and very versatile experimental system to investigate the functional relationship between miRNAs and their targets relevant to oral cancer in vivo. Indeed, our current findings successfully support this idea of *Drosophila* as a novel in vivo platform.

Our findings also suggest advantages of using multiple platforms spanning diverse phyla when validating miRNA targets that are less likely to be listed as top candidates by the public database. Despite a significant degree of sequence homology between human and *Drosophila* miR-31 (Figure 2A), *wntless* (*wls*) was recognized as one of the top miR-31 targets in *Drosophila*, but rarely in the human database (Tables S1 and S2). Importantly, our combinatorial approach using both *Drosophila* wing discs and cultured human oral cancer cells successfully demonstrate *WLS* as a common target of miR-31 in both platforms (Figure 2, Figure 3 and Figure 4). Most computational algorithms perform their prediction of miRNA targets based upon complementary base pairing between miRNA and mRNA, with the accuracy of less than 70% according to a recent report [102]. Together with our results, it raises a concern of relying solely on the prediction results derived from computational algorithms in a single species when studying the miRNA-target relationship and may thus demand a diverse array of screening methods using multiple experimental platforms for validation. It should be noted that a discrepancy in initial prediction results may stem from a relatively low similarity between human and *Drosophila WLS* mRNAs restricted to a subregion, in comparison with higher overall sequence identity in their protein products (41%).

The early in vitro studies on miR-31 suggest its role in acquisition of immortality in normal oral keratinocytes, thus progressing into malignant tumors in its early stage [28], or as a responder when oncogenic processes are initiated by other factors such as epidermal growth factor [30]. These results are in contrast to our findings of miR-31 playing a suppressive role in tissue growth (Figure 2). Furthermore, our study clearly demonstrates miR-31-induced downregulation of the cell-cycle regulatory molecules, including the well-established proto-oncogenes *cyclin D1 (CCND1*) and *c-MYC* (Figure 5A–D), presumably linked to a delay in the cell cycle of oral cancer cells (Figure 5E,F). In line with our data, a recent analysis on recurrent oral leukoplakia, a potentially malignant lesion, has revealed a pro-apoptotic role of miR-31 in limiting the progression of oral leukoplakia lesions into malignant tumors via downregulation of fibroblast growth factor 3 [103]. Together with the clinical cancer genome analyses (Figure 1), our finding thus provides another layer of experimental evidence to support the idea of miR-31 as a tumor suppressor. It should be noted that we had to elevate the expression level of miR-31 in the oral cancer cells to visualize its effect on *WLS* and cell cycle regulation due to the low level of its endogenous expression. Whether the functional interaction between miR-31 and *WLS* would also be linked to tumorigenesis and progression of oral cancer in a clinical setting awaits further investigations.

WLS is an important regulator of Wnt signaling [90,91], a well-known pathway of which dysregulation has been linked to tumorigenesis and progression of various types of malignant tumors [7,8]. Recent studies on oral cancer indicate both the canonical and non-canonical Wnt pathways as targets of multiple miRNAs, including miR-329, miR-410 and miR-21 [21,104]. Here we present another example of miR-dependent regulation of the Wnt signaling pathway that may play a role in cell cycle regulation of oral cancer cells, thus potentially affecting the rate of tumor growth. Our further analyses on the RNAseq data available in a TCGA collection, consisting of 236 control and 145 OSCC tumor samples (Table S3), indicate a prominent upregulation of *WLS* in oral cancer patients that coincided with a lower survival rate (Figure S6). Despite the lack of differential expression between the controls and patients deduced from the aforementioned TCGA collection, the lower expression of *CCND1* was also correlated with a better prognosis in patients, as indicated by a higher survival rate (Figure S7). These results may further provide clinical significance to our finding of miR-31-dependent regulation of WLS and its downstream targets.

It should be noted that our data on the level of some canonical Wnt signaling molecules, such as GSK3β and β-catenin, were inconclusive, with a decrease in their expression only in a subset of cultures (Figure S3), albeit with consistent downregulation of their transcriptional targets, *CCND1* and *c-MYC* (Figure 5). These may be in part related to the variable amount of miR-31 transfected or expressed in each culture. Considering the role of WLS in Wnt trafficking to induce activation of the downstream effectors [90,91], transcriptional or translational regulation of the Wnt signaling molecules induced by miR-31 may not be apparent for detection. Alternatively, it is possible that these downstream targets may be regulated in parallel by miR-31 in a Wnt-independent manner. While our data mostly center around the canonical Wnt pathway, WLS may also influence the activity of the non-canonical Wnt pathways, independent of GSK3β and β-catenin. Further systematic analyses are thus required to explore the possibility of miR-31-dependent control of cell cycle regulators beyond the canonical Wnt signaling pathway.

## 4. Conclusions

In summary, we demonstrate a successful implementation of a mammal-to-*Drosophila*-to-mammal approach to decipher the potential functional interaction between oral cancer-related miRNAs and their putative targets by establishing bi-directional platforms consisting of human oral cancer cells in vitro and *Drosophila* tissues in vivo. To our best knowledge, this is the first report to provide an example of this combinational approach applied to oral cancer, one of the intractable human malignancies with poor prognosis. Our study will provide a unique opportunity to further explore the relationship between miRNAs and various types of human cancer beyond oral malignancy, with the aim of developing more effective treatment options to cure these devastating illnesses.

## 5. Materials and Methods

### 5.1. Fly Stocks

All crosses and stocks were kept on a standard medium at 24 °C with a humidity between 45% and 60%. All fly stocks used in our study were acquired from the Bloomington Drosophila Stock Center (Bloomington, IN, USA), including w^*^; *PBac{UAS-mir-31a.S}VK00037/CyO*, *w^1118^*; *P{UAS-LUC-mir-31a.T}attp2*, *w^1118^*; *P{UAS-LUC–mir-31b.T}attP2*, w^*^; *P{GawB}459.2* and y^1^w^*^; *P{GawB}ush^MD751^*. The *459.2-* and *ush-GAL4* drivers were used for expression of the *UAS-miRNA-31* transgene in the imaginal wing discs of the third instar larvae.

### 5.2. Image Analysis

Larval wing discs were dissected from the wondering third instar larvae in HL3.1 saline. They were fixed in PBS containing 3.7% formaldehyde and mounted in DAPI-conjugated Vectashield^®^ Antifade Mounting Media (Vector Laboratories, Burlingame, CA, USA) for imaging. The images of the wing discs were taken with an Olympus microscope EX51 (Olympus, Center Valley, PA, USA) and processed with Adobe^®^ Photoshop CS6 (Adobe Corporation, San Jose, CA, USA). The overall size of the individual wing discs was measured using the ImageJ package (National Institutes of Health, Bethesda, MD, USA).

### 5.3. Cell Culture

The human oral squamous cell carcinoma (OSCC) cell lines, including SAS and OSC20, were maintained in Dulbecco’s Modified Eagle’s Medium (DMEM) and Ham’s nutrient mixture F12 (Hyclone, Logan, UT, USA) supplemented with 10% fetal bovine serum (FBS, GIBCO, ThermoFisher Scientific, Waltham, MA, USA) and 100 units/mL of penicillin–streptomycin (Invitrogen, ThermoFisher Scientific, Waltham, MA, USA). For generating stable cell lines expressing miR-31, it was subcloned into the pMSCV-puro retroviral vector (Takara Bio Inc, Kusatsu, Japan). The prepared vector (10 μg) was then transfected into Phoenix^TM^ packaging cells (ThermoFisher Scientific). Viral supernatants were collected 48 h later and filtered for further processing. Cells expressing the ectopic miR-31 were positively selected by an application of puromycin at the concentration of 2 μg/mL (Invitrogen, ThermoFisher Scientific). All cells were maintained at 37 °C in a humidified incubator with 5% CO_2_.

### 5.4. Transfections of miR-31 Mimic

For overexpression of miR-31 mimic and miR-31 inhibitor in OSCC cells, we designed short double-stranded RNAs as miR-31 mimic and inhibitor with the following sequence: UGCUAUGCCAACAUAUUGCCAU for miR-31 mimic and AGGCAAGAUGCUGGCAUAGCU for miR-31 inhibitor (Bioneer Co, Ltd., Daejeon, South Korea). Corresponding scrambled miRNA was also created and used as controls. Transfection was carried out using Fugene^®^ 6 Transfection Reagent (Promega, Madison, WI, USA) and X-tremeGENE^™^ siRNA Transfection Reagent (Sigma-Aldrich Inc., St. Louis, MO, USA) according to the manufacturer’s instructions. The culture medium was replaced with fresh DMEM and Ham’s F12 supplemented with 10% FBS at 6 h following transfection. Cells were then further incubated for additional 42 h, washed with PBS twice and subjected to subsequent qRT-PCR and western blot analyses.

### 5.5. Reverse Transcription Polymerase Chain Reaction (RT-PCR)

A total of five to ten wing discs were homogenized in 100 μL of PBST (PBS with 0.1% Triton x-100). Total RNA was extracted from imaginal wing discs of the third instar *Drosophila* larvae using the TRIzol reagent (Invitrogen, ThermoFisher Scientific), followed by a synthesis of cDNA from 2 μg of extracted RNA using TOPscript^TM^ cDNA synthesis kit (Enzynomics, Inc., Daejeon, Korea), according to the manufacturer’s instructions. A standard RT-PCR analysis was performed using PrimeScript RT Master Mix (Takara Bio Inc.), according to the manufacturer’s instructions. Mir-X^TM^ miRNA First-Stand Synthesis Kit (Qiagen Inc., Germantown, MD, USA) was used for amplification of the coding region of mature miRNAs. Each RT-PCR reaction was followed by gel electrophoresis and visualization using Multiple Gel DOC system (Fuji Photo Film Co., Ltd., Tokyo, Japan).

### 5.6. Quantitative Measurement of miR-31 and Its Target Transcripts

The real-time quantitative RT-PCR reaction (qRT-PCR) was performed using TOPreal^TM^ SYBR Green qPCR master mix (Enzynomics, Inc.). The reaction cycles were initiated with a 10 min-long step of denaturation (95 °C), followed by 40 cycles of reactions each consisting of denaturation (95 °C for 15 s), annealing (60 °C for 30 s) and extension steps (72 °C for 30 s). The quantification of each transcript was done using the 7500 Real-Time PCR Instrument System (Applied Biosystems, ThermoFisher Scientific, Waltham, MA, USA). The mRNA levels of human *GADPH* and *Drosophila actin* were measured and served as internal controls for normalization. All RT-qPCR experiments consisting of triplicates were repeated at least three times for statistical analyses. The primer sets used include *Drosophila wls*, CAGGTGTTGTGCTTCCTGCT as a forward primer and CCATCTTTGTTGAATCCTGCTCC as a reverse primer; *Drosophila actin*, CCACACCGTCCCCATCTATG as a forward primer and AGTCCAGGGCAACATAGCAC as a reverse primer; human WLS, TGGGATTCTGCTCGTGTTC and TCTACAAGTTGACCCGCAAG as forward primers and TCTTGTGATGGTTCTTACGGG and CAGCTAAGAGCCATGAGGC as reverse primers; human *WNT3A*, CCAAGTCGAGGGCAAACAGAA as a forward primer and TGGATCGCTGGGTCCATGTA as a reverse primer; *human CCND1*, AACTACCTGGACCGCTTCCT as a forward primer and CCACTTGAGCTTGTTCACCA as a reverse primer; *human c-MYC*, AATGAAAAGGCCCCCAAGGT as a forward primer and GTCGTTTCCGCAACAAGTCC as a reverse primer; and *human GAPDH*, CACCATCTTCCAGGAGCGAG as a forward primer and GACTCCACGACGTACTCAGC as a reverse primer. The primers for human miR-31 were purchased from Antibody-Antibodies.com (ABM-MPH01329 and ABM-MPH-02458) (Gentaur Molecular Products, Kampenhout, Belgium).

### 5.7. Western Blot Analysis

OSCC cells were lysed in RIPA buffer (pH 7.6, 50 mM Tris HCl, 300 mM NaCl, 0.5% Triton X-100, 2 μL/mL aprotinin) (Elpis-Biotech, Inc., Daejeon, Korea) mixed with 2 mM of PMSF and 2 μL/mL of leupeptin. The lysate was subjected to a Pierce BCA protein assay (ThermoFisher Scientific) for measuring the amount of protein, then separated in a SDS-PAGE gel and transferred onto nitrocellulose membranes (MilliporeSigma, Burlington, MA, USA), with the subsequent blocking step using 3% BSA. The primary antibodies used include: β-Actin, β-catenin, p-β-catenin, GSK3β, p-GSK3β (Santa Cruz Biotechnology, Dallas, TX, USA), Wntless (WLS) (Abcam, Cambridge, MA, USA) and Wnt3a (Cell Signaling Technology, Danvers, MA, USA). Chemiluminescence was detected using the WesternBright^TM^ ECL (Advansta Inc., San Jose, CA, USA) and WesternBright^TM^ Sirius kit (Advansta Inc.) together with the secondary antibody (Cell Signaling Technology). The signal intensity of each protein band was then quantified using a LAS 3000 (Fuji Photo Film Co., Ltd.).

### 5.8. Luciferase Assay

OSC20 cells were seeded on 24-well plates, into which 4X CSL reporter plasmids (0.5 μg each) were separately transfected. These cells were lysed in a passive lysis buffer after 48 h of transfection. Target promoter-driven firefly luciferase activity was then measured with a Dual-Luciferase Reporter Assay System (Promega) and normalized to that of the *Renilla* control. All experiments were repeated three times for a statistical analysis.

### 5.9. Flow Cytometry Analysis

Cells were cultured with the confluency of 70~80% in 60 mm culture dishes for 24 h and exposed to delphinidin for additional 24 h. The cells were then harvested using trypsinization, centrifuged at 3000 rpm for 5 min, fixed with ice-cold 70% ethanol and stored at 4°C overnight. The fixed cells were washed in PBS solution with 1% bovine serum albumin and re-suspended in a staining buffer containing propidium iodide (PI) (1 mg/mL) and RNase A (50 mg/mL), followed by an incubation at 4°C for 30 min. These cells were then stained with PI (50 μg/mL). The fluorescence from stained cells was measured and analyzed for the fractions of cells at different cell cycle phases using CYTOMICS FC500 flow cytometer system (Beckman Coulter, Brea, CA, USA).

### 5.10. Gene Expression and Survival Analysis of TCGA Collection

The RSEM normalized RNAseq data of OSCC were downloaded from Broad GDAC Firehose (https://gdac.broadinstitute.org/) for TCGA-HNSC. We selected neoplasmic subdivisions of TCGA-HNSC using the following anatomic sites of primary tumors: lip, tongue, floor of mouth, buccal mucosa, hard palate, oropharynx, tonsil and oral cavity (unspecified), consisting of 236 control and 145 OSCC tumor samples. The WLS and CCND1 expression levels in the control and OSCC samples were compared using a Mann–Whitney–Wilcoxon test with Bonferroni correction based on the statannot python package (statannot version 0.2.2 and python version 3.7.1, Python Software Foundation, 2020). The survival analyses were then performed using the lifelines python package (lifelines version 0.24.0, Python Software Foundation, 2020).

### 5.11. Statistical Analysis

Comparisons between two independent groups were made by unpaired two-tailed Student’s *t*-tests. For multiple comparisons among three groups or more, we adopted one-way analysis of variance (ANOVA) followed by post-hoc Tukey’s post-hoc tests. For an analysis of the RNAseq data of OSCC, a Mann–Whitney–Wilcoxon test was conducted with Bonferroni correction (see above). All statistical analyses were performed using the OriginPro 2020b (OriginLab Corporation, Northampton, MA, USA) and Phython packages (Python Software Foundation, 2020). A *p* value less than 0.05 was considered statistically significant.

## Figures and Tables

**Table 1:** Differential expression of microRNAs (miRNAs) in oral cancer cell lines or tumor tissues. miRNAs showing differential expression profiles in oral cancer cell lines or tumor tissues reported in previous studies are listed, with the change in their expression levels indicated.

miRNA ID	Expression Profile of miRNAs
hsa-let-7d	*Downregulated* [25]
hsa-miR-10b	Upregulated [26]
hsa-miR-21	Upregulated [21,27]
hsa-miR-31	Upregulated [28,29,30,31]
hsa-miR-34a	*Downregulated* [32]
hsa-miR-141/200/429	*Downregulated* [33]
hsa-miR-143	*Downregulated* [34]
hsa-miR-196b	Upregulated [35]
hsa-miR-214	Upregulated [36]
hsa-miR-218	*Downregulated* [37,38]
hsa-miR-223	Upregulated [39]
hsa-miR-340	*Downregulated* [40]
hsa-miR-375	*Downregulated* [41,42]
hsa-miR-494	*Downregulated* [43,44]

**Table 2:** Differential expression of miR-31 in various types of cancer. Previous results demonstrating the up- or down-regulation of miR-31 are summarized for a subset of malignant tumors, with the change in their expression levels indicated.

Family/Cluster Cancer	Expression Profile of miR-31
Prostate cancer	*Downregulated* [45,46,47]
	Upregulated [48]
Liver cancer	*Downregulated* [49,50]
	Upregulated [51,52]
Lung cancer	*Downregulated* [53,54]
	Upregulated [55,56,57,58]
Breast cancer	*Downregulated* [59]
Gastric cancer	*Downregulated* [60,61,62,63,64]
Glioblastoma	*Downregulated* [65,66,67]
Melanoma	*Downregulated* [68,69]
Leukemia	*Downregulated* [70]
Colon/Colorectal cancer	Upregulated [71,72,73,74,75,76]
Cervical cancer	Upregulated [77,78,79]
Head and neck cancer	Upregulated [80]
Esophageal cancer	Upregulated [81]
Thyroid cancer	Upregulated [82]
Pancreatic cancer	Upregulated [83]

## Supplementary Materials

The following are available online at https://www.mdpi.com/1422-0067/21/19/7232/s1, Figure S1: Overexpression of miR-31 using the GAL4-UAS system in Drosophila wing discs, Figure S2: Three different types of matches between a miRNA and its predicted mRNA targets categorized by TargetScan, Figure S3: Putative targeting of human WLS mRNA by hsa-miR-31-5p, Figure S4: The effect of transfected and stably expressed miR-31 on the level of WLS and CCND1 in OSCC cells, Figure S5: Expression of Wnt pathway components in OSCC cells following transient transfection of miR-31, Figure S6: Differential expression of WLS and its correlation with the survival rate in oral cancer patients, Figure S7: Differential expression of CCND1 and its correlation with the survival rate in oral cancer patients, Table S1: Putative targets of dme-miR-31a/b predicted by TargetScanFly, Table S2: Putative targets of hsa-miR-31 predicted by TargetScanHuman, Table S3: Description of variables extracted from the RSEM RNAseq analyses on TCGA-OSCC.

## Abbreviations

OSCC	oral squamous cell carcinoma
WLS	Wntless
miR-31	microRNA-31
CCND1	cyclin D1
