# Supplementary material for: MicroRNA-31 Regulates Expression of Wntless in Both Drosophila melanogaster and Human Oral Cancer Cells
Source: Int J Mol Sci. 2020 Sep 30;21(19):7232. doi: 10.3390/ijms21197232 (PMC7582764; doi:10.3390/ijms21197232)
Supplement: Supplementary file 1 [file ijms-21-07232-s001.pdf]

MicroRNA-31 regulates expression of *Wntless* in both *Drosophila melanogaster* and human oral cancer cells

Ji-Eun Jung[1,2], Joo-Young Lee[3], In-Ryoung Kim[4], Sang-Mee Park[4], Hye-Ryoun Park[1,2,3,4] and Ji-Hye Lee[1,2,3,4*]

[1]Department of Life Science in Dentistry, [2]BK21Plus Project, [3]Dental and Life Science Institute, [4]Department of Oral Pathology, [5]Department of Anatomy, School of Dentistry, Pusan National University, Yangsan 50612, Korea

***Corresponding Author:**

Ji-Hye Lee

Department of Oral Pathology, School of Dentistry,

Pusan National University

49 Pusandaehak-Ro, Mulgeum-Eup

Yangsan-Si, Kyoungsangnam-Do 50612

Korea, Republic of

Phone: +82-51-510-8259

E-mail: jihyelee@pusan.ac.kr

SUPPLEMENTARY MATERIALS (3 Figures and 2 Tables)

Supplementary Figures

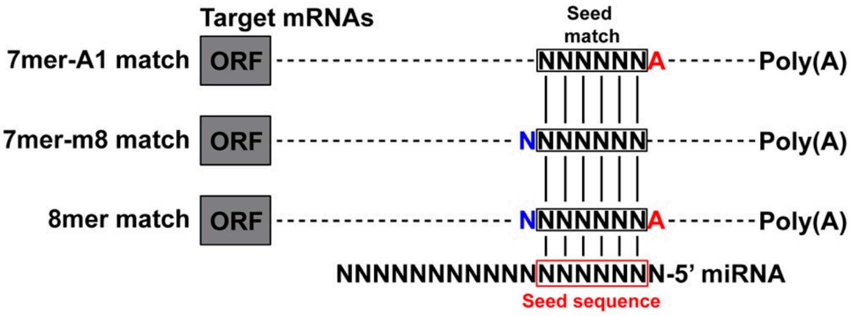

Supplementary Figure S1. Three different types of matches between a miRNA and its predicted mRNA targets categorized by TargetScan.

In addition to the core seed outlined with boxes, additional nucleotide can further be matched either at the position 8 (7mer-m8), the additional "A" preceding the seed sequence (7mer-A1), or both (8mer).

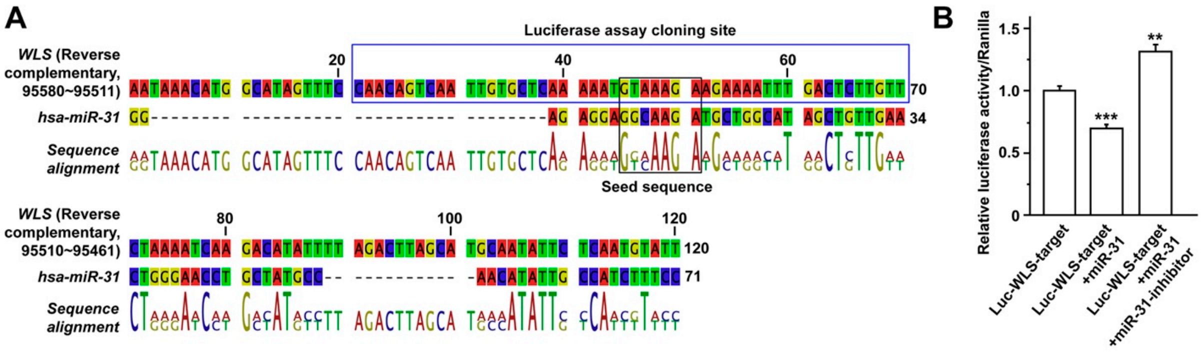

Supplementary Figure S2. Putative targeting of human WLS mRNA by hsa-miR-31-5p.

(A) The 3'-UTR regions of human *WLS* mRNA are probed for its putative match with the seed sequence of mature hsa-miR-31-5p. The cloning site for a luciferase assay is indicated with a blue

box. (B) The relative luciferase activity is compared among OSC20 cells transfected with 1) a construct containing *WLS* 3'UTR sequence (blue box in A) fused with luciferase domain (Luc-WLS target), 2) a *WLS* 3'UTR-containing construct as well as a miR-31 mimic (Luc-WLS target+miR-31), or 3) a *WLS* 3'UTR-containing construct, a miR-31 mimic and a synthetic single-stranded miR-31-inhibitor oligonucleotide complementary to mature miR-31 (Luc-WLS target+miR-31+miR-31-inhibitor). Mean± SEM indicated. **, *P*<0.01 and ***, *P*<0.001.

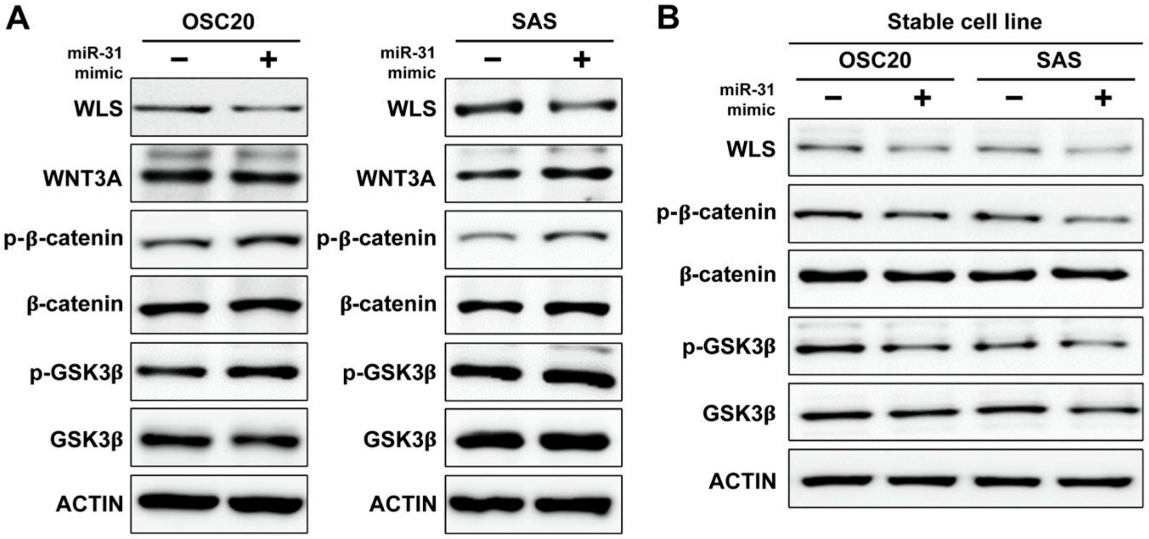

Supplementary Figure S3. Expression of Wnt pathway components in OSCC cells following transient transfection of miR-31.

(A and B) The protein levels of Wnt pathway components are shown in a western blot analysis 1) before and after transfection of miR-31 mimics in SAS and OSC20 cells (A) or 2) in a stable miR-31-expressing SAS and OSC20 cell lines (B).

Supplementary Tables

Table S1. Putative targets of dme-miR-31a/b predicted by TargetScanFly

Target Gene ID	Gene symbol	Types of target sites			
		8mer	7mer-m8	7mer-A1	Total
CG11247	*CG11247*	1	0	1	2
CG6210	*wls*	0	1	0	1
CG10079	*Egfr*	1	0	0	1
CG6634	*mld*	1	0	0	1
CG31240	*repo*	1	0	0	1
CG13209	sha	1	0	0	1
CG1136	*CG1136*	1	0	0	1
CG16947	*CG16947*	1	0	0	1
CG17390	*CG17390*	0	0	1	1
CG33106	*mask*	0	0	2	2
CG7852	*CG7852*	0	0	1	1
CG13287	*CG13287*	0	0	1	1
CG30429	*CG30429*	0	1	0	1
CG31243	*cpo*	0	1	0	1
CG32346	*E(bx)*	0	1	0	1
CG1864	*Hr38*	0	1	0	1
CG11770	*lin*	0	1	0	1
CG4816	*qkr54B*	0	1	0	1
CG4316	*Sb*	0	1	0	1
CG14223	*CG14223*	0	1	0	1
CG14767	*CG14767*	0	1	0	1
CG14837	*CG14837*	0	1	0	1
CG18641	*CG18641*	0	1	0	1
CG32446	*CG32446*	0	1	0	1

Different sites of each gene putatively targeted by *Drosophila* miR-31 (dme-miR-31a/b) are listed. The target sites determined by TargetScanFly (ver. 7.2) are categorized into three types: 7mer-A1, 7mer-m8 and 8mer sites (http://www.targetscan.org/docs/7mer.html; Supplementary Figure S1).

Table S2. Putative targets of hsa-miR-31 predicted by TargetScanHuman

Target Gene Symbol	Gene name	Types of target sites			
		8mer	7mer-m8	7mer-A1	Total
RNF144B	ring finger protein 144B	0	0	1	1
RSBN1	round spermatid basic protein 1	1	1	0	2
SH2D1A	SH2 domain containing 1A	1	0	0	1
AK4	adenylate kinase 4	1	1	0	2
PAX9	paired box 9	1	0	0	1
LPP	LIM domain containing preferred translocation partner in lipoma	0	0	2	2
PRKCE	protein kinase C, epsilon	1	0	0	1
NR5A2	nuclear receptor subfamily 5, group A, member 2	1	1	0	2
TMEM145	transmembrane protein 145	1	0	0	1
ARHGEF2	ho/Rac guanine nucleotide exchange factor (GEF) 2	0	0	1	1
WDR5	WD repeat domain 5	1	0	0	1
PSMB11	proteasome (prosome, macropain) subunit, beta type, 11	1	0	0	1
PEX5	peroxisomal biogenesis factor 5	1	1	0	2
FGF7	fibroblast growth factor 7	1	0	0	1
SYDE2	synapse defective 1, Rho GTPase, homolog 2 (C. elegans)	2	0	0	2
PDZD2	PDZ domain containing 2	1	0	0	1
SSH1	slingshot protein phosphatase 1	1	0	0	1
TFRC	transferrin receptor	1	0	0	1
IL34	interleukin 34	1	0	0	1
CIAPIN1	cytokine induced apoptosis inhibitor 1	1	0	0	1
PC	pyruvate carboxylase	1	0	0	1
CRYBG3	beta-gamma crystallin domain containing 3	1	0	0	1
EGLN3	egl-9 family hypoxia-inducible factor 3	1	0	0	1
TBXA2R	thromboxane A2 receptor	1	0	0	1

Different sites of each gene putatively targeted by human miR-31 (hsa-miR-31) are listed. The target sites determined by TargetScanHuman (ver. 7.2) are categorized into three types: 7mer-A1, 7mer-m8 and 8mer sites (http://www.targetscan.org/docs/7mer.html; Supplementary Figure S1).
